# Peripheral Nerve Innervation in Bilateral Cleft Hand Syndrome Elucidated by Ultrasound

**DOI:** 10.3389/fneur.2022.857363

**Published:** 2022-05-20

**Authors:** Pietro Falco, Steven Hovius, Nens van Alfen

**Affiliations:** ^1^Department of Human Neuroscience, Sapienza University, Rome, Italy; ^2^Department of Plastic Surgery, Radboud University Medical Center, Nijmegen, Netherlands; ^3^Department of Neurology and Clinical Neurophysiology, Donders Institute for Brain, Cognition and Behavior, Radboud University Medical Center, Nijmegen, Netherlands

**Keywords:** cleft hand syndrome, peripheral nerve anatomy, nerve ultrasound, reconstructive surgery, congenital malformations

## Abstract

Bilateral cleft hand syndrome is a rare congenital malformation with complex anatomy. Previous reports have mainly focused on the description of bone and soft tissue abnormalities, but information about innervation is scarce. Knowledge of the peripheral nerve anatomy is helpful for surgical treatment, optimizing the reconstruction, and preventing iatrogenic damage. Following clinical assessment and conventional radiologic imaging, we used high-resolution ultrasound of both hands and forearms to image the peripheral nerves in a patient with severe bilateral cleft hand syndrome. The patient presented with two ulnar digits, a deformed thumb on the right, and a rudimentary thumb appendage on the left. In keeping with the tissue elements present and absent, we found a severe bilateral nerve size reduction of the median nerves, sparing the anterior interosseous nerve fascicles. The radial nerve and end branches were intact, and a slightly smaller ulnar nerve was found that ended in two digital branches to a single digit. Our study shows that in cleft hand syndrome the peripheral nervous system anatomy exactly reflects the presence and absence of the corresponding muscle and skin innervation areas. This information is helpful for planning a surgical-reconstructive approach and suggests a potential role for nerve ultrasound in the assessment of complex limb malformations.

## Introduction

**Figure 1:**
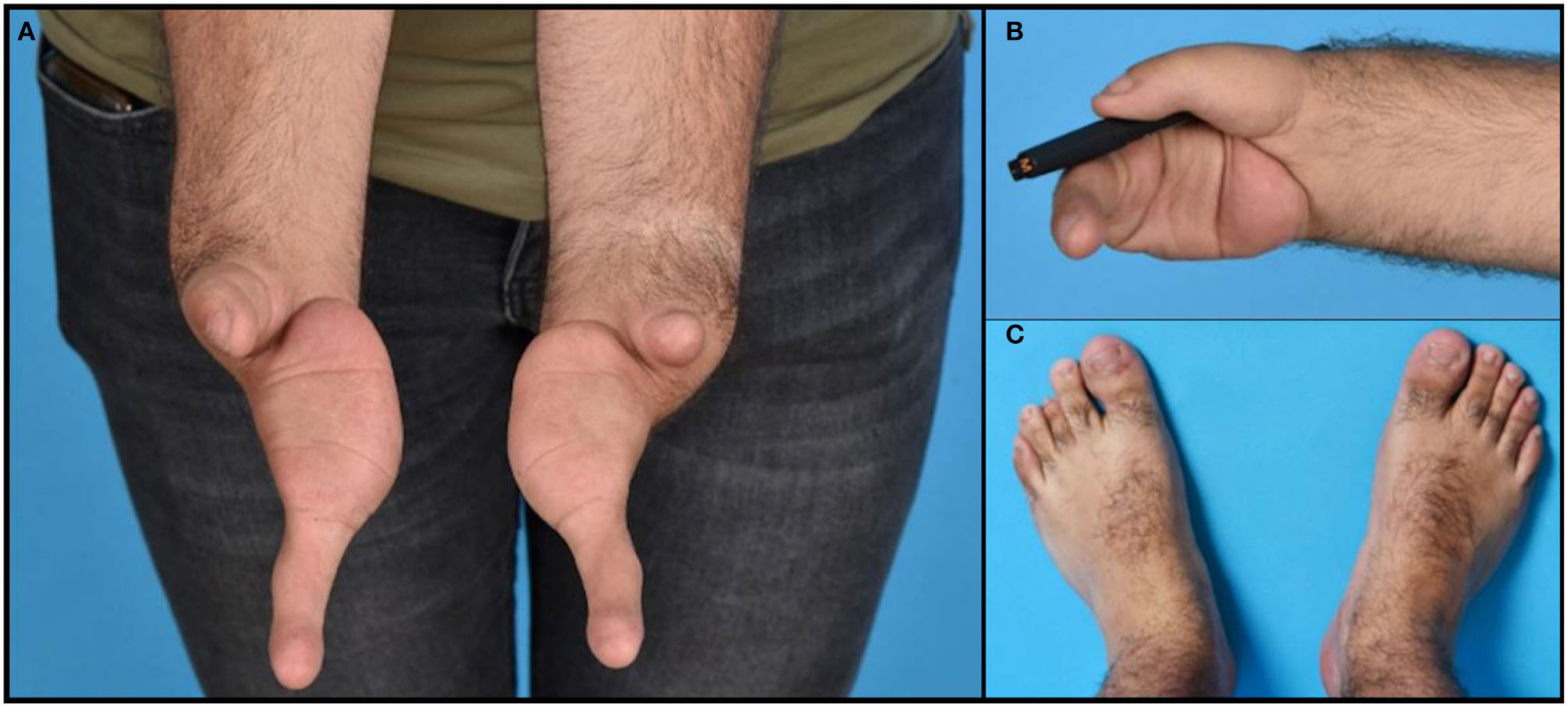
Photographs of the hands **(A)**, feet **(C)**, and an example of functional use of the right hand holding a pen **(B)**.

A 25-year-old man presented to us with, in our opinion, a cleft hand syndrome. He had a bilateral complete absence of three fingers and metacarpus, with a bilateral long “ulnar finger,” a hypoplastic thumb with a highly unstable carpometacarpal joint and thenar hypoplasia on the right side, and only a short soft tissue stump at the end of radius at the radial side (Figure 1A). The patient sought surgical-reconstructive counseling because of the social repercussions and emotional distress caused by his malformation.

Cleft hand syndrome, or ectrodactyly, is a congenital malformation defined as a longitudinal deficiency with suppression of bone and soft tissue in the central elements of the hand, including the index, middle, and ring fingers (1). In the updated Oberg-Manske-Tonkin (OMT) classification (2, 3) they are classified as abnormal axis formation/differentiation-handplate (B) in the unspecified axis (4), complex (iii), and subsequently cleft hand (B.4.iii.c).

Reports of patients with cleft hands, both from cadaveric dissections and imaging studies, have mainly focused on descriptions of bone and soft tissue, such as muscle and tendons (4). However, we have not been able to find information regarding peripheral nerve innervation patterns. This information is desirable to optimize planning for surgical reconstruction. Nerve ultrasound (5) has become a very useful method for the diagnostic evaluation of distal peripheral nerve injuries, because of its higher resolution and sensitivity compared to other imaging techniques such as MRI (6). Ultrasound is an attractive addition to the workup because of its availability, cost, and ease of use at the bedside and intraoperatively. Its use in pre-surgical planning is growing and it has demonstrated a high capability to prevent iatrogenic peripheral nerve injuries (7). The purpose of this report is to describe the peripheral nerve innervation of both forearms and hands using high-resolution ultrasound in a patient with severe bilateral cleft hands.

## Methods

The patient underwent clinical assessment including photographic documentation, standard X-ray studies of the hands, and MRI of the forearms to assess the bony and musculotendinous anatomy. As a part of the pre-surgical workup, bilateral high-resolution nerve ultrasound of the superficial radial, ulnar, and median nerves bilaterally was performed, following the nerves from the proximal forearm to the distal branches, to better define the anatomical characteristics of the peripheral nervous system in this rare type of congenital malformation. We used a Canon Aplio i800 ultrasound machine (Canon Medical Systems Cooperation, Tokyo, Japan) with a high-frequency 8–24 MHz linear matrix array probe with a 38 mm footprint, using a specifically created “nerve” preset set to 19 MHz center frequency, with focus and depth settings to match the course of the nerves during the examination.

## Results

### Clinical Assessment

The ulnar finger on each hand had three phalanges and a metacarpal resting on the ulnar pillar of the carpus. On the ulnar side of the hand, the soft tissues of the palm were superfluous. The skin and nails of the fingers were intact (Figure 1A). The hand on the rudimentary carpus' was very mobile to unstable in all directions. The ulnar finger on both hands flexed only at the thumb carpometacarpal (CMC) joint. The patient was right-handed and had a key grip with the hypoplastic thumb to the proximal phalanx of the ulnar finger, although with little strength (Figure 1B). His ulnar finger on both sides was hypermobile in the CMC joint but hardly moved in the interphalangeal joints. No defects in strength or sensation were found for all the anatomical structures that were present and clinically accessible.

There was no history of congenital malformations in the patient's family. His parents were consanguineous with a second degree of kinship. He was exposed to an unknown drug during the first 3 months of intrauterine life. In addition to the hand deformities, the patient also presented with the following congenital malformations: bilateral hip dysplasia, hypospadias, gonadal retention (treated with orchidopexy), and hypopituitarism with hypocortisolism and hypoaldosteronism, which was treated with hydrocortisone and fludrocortisone replacement therapy. He also suffered from Perthes disease. His face and lower extremities demonstrated a shorter 3rd toe on the left foot (Figure 1C) but no further malformations. His cognitive function was normal, and he was partially independent with regard to employment, working in his parents' restaurant. During a previous workup, Sanger sequencing was performed of the TP63 gene associated with split hand and foot malformation, and subsequent whole-exome sequencing was performed, but no genetic abnormalities were detected.

According to the DAST functional classification of cleft hand (D = digits missing, A = associated anomalies in the hand, S = site of cleft, T = functional state of the thumb), the patient presented a D-3 (three or more), A-0, S-1 (1 = central), T-4 (4 is hypoplastic) score on the right side and a D-4, A-0, S-1, T-5 (floating thumb or agenesis) score on the left side. This score implied a severe cleft type that would most likely need reconstruction with a vascularized toe transfer on the left (8).

### X-Ray and MRI Studies

**Figure 2:**
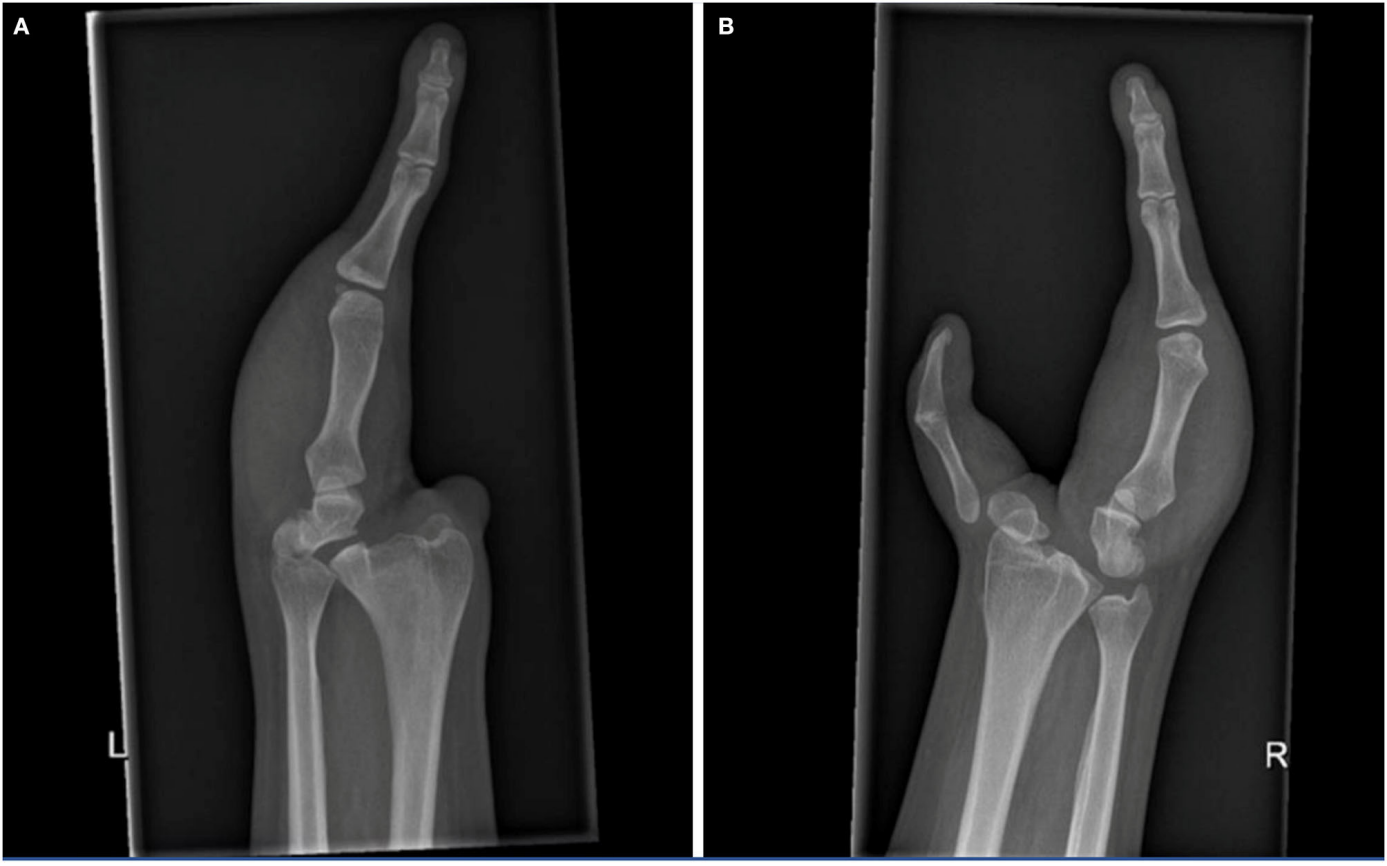
X-rays of the left **(A)** and right hand **(B)**, show the osseous structures in this patient with cleft hands. Note the symphalangism of the right thumb bones.

For the X-ray (Figures 2A,B) and MRI of both hands and forearms, there was a display of a dysplastic aspect with the irregular articular surface of both distal radius and ulna of both forearms. The carpus on the right side showed the presence of a rudimental trapezium fused with a rudimental scaphoid on the radial side and the ulnar side a malformed hamate, triquetrum, and pisiform. On the left side, the carpus existed of a dysplastic scaphoid bone on the radial side and a hamate fused with a rudimental triquetrum and a pisiform bone on the ulnar side. On both sides, the ulnar finger consisted of three phalanges and a metacarpal bone with abnormal CMC joints. On the right side, the thumb was hypoplastic, the first metacarpal fused with the corresponding phalanges. The proximal end of the first metacarpal was subluxed with an abnormal CMC1 joint. The ulnar soft tissue comprised skin and fat with poorly distinguishable intrinsic muscles and tendons. All forearm muscles and tendons were present and appeared normally formed, although we were unable to assess whether muscle volumes were slightly decreased, due to the bilateral pathology.

### High-Resolution Ultrasound

**Figure 3:**
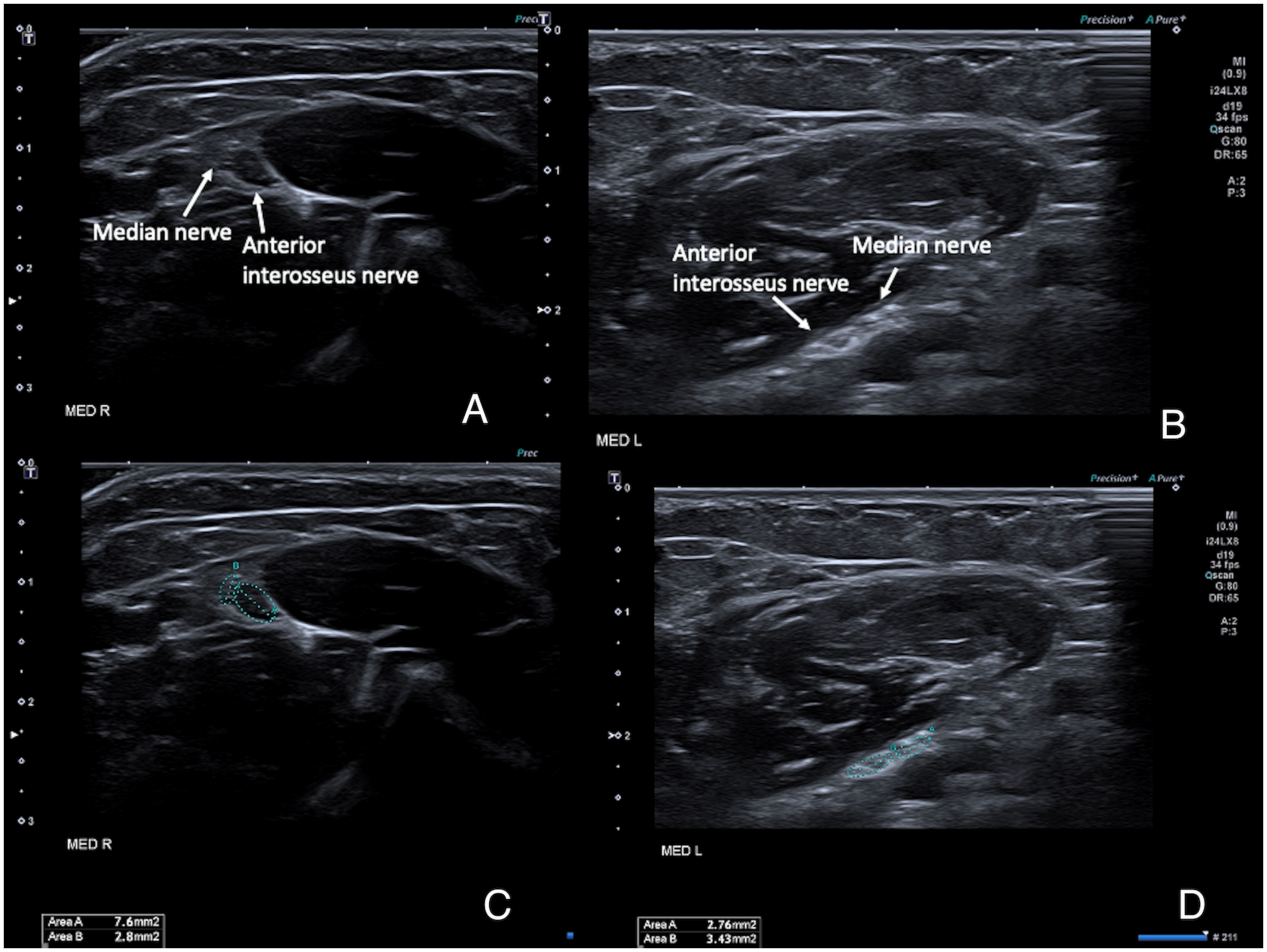
High-resolution ultrasound images of the right **(A,B)** and left **(C,D)** median nerves at the level of the elbow, with the nerves running between the deep and superficial head of the pronator teres muscle. **(A,C)** show the anatomical configuration, with an unusually small median nerve proper and a comparatively larger anterior interosseus nerve, and even more pronounced on the right (where a thumb was present) which is the opposite of normal. **(B,D)** show the same images with the cross-sectional area measurements superimposed. Normal adult average reference cross-sectional areas (CSAs) in this region is around 7–9 mm^2^ for the median nerve proper at the elbow level and 3–4 mm^2^ for the anterior interosseus nerve.

**Figure 4:**
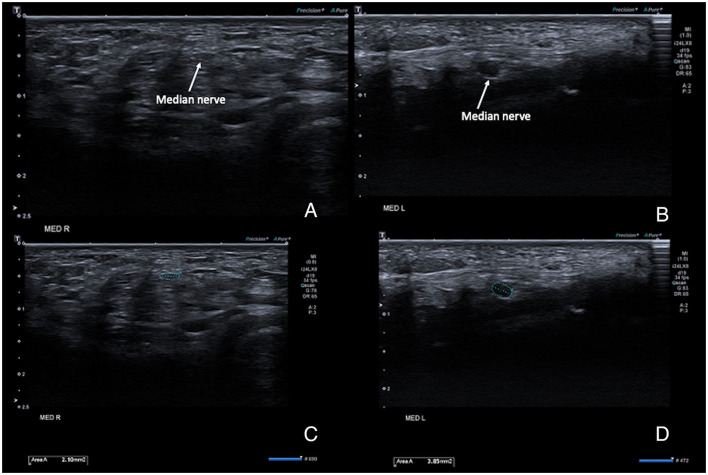
High-resolution ultrasound images of the right **(A,B)** and left **(C,D)** median at the level of the carpal tunnel inlet at the wrist. **(A,C)** show the anatomical configuration, with a very small nerve size bilaterally, but a comparatively hypoechogenic and enlarged aspect of the nerve on the left. **(B,D)** show the same images with the cross-sectional area measurements superimposed. Normal adult average reference CSAs of the median nerve at the level of the carpal tunnel inlet vary from 6–9 mm^2^.

Scanning the forearm muscles and the soft tissues in the wrist regions confirmed the MRI appearance of muscles and tendons. The median nerve on both sides ran a normal anatomical course in the arm and forearm, but with a pronounced reduction of the cross-sectional area (CSA) of the median nerve proper, while the anterior interosseus nerve (AIN) fascicles had a normal size before and after branching. Ultrasonography could be seen as an inversion of the normal CSA ratio between the two nerve bundles at the elbow level, with an AIN branch that was larger than the main median nerve branch (Figures 3A–D). After the branching of the AIN, the small median nerve properly followed a normal course between the deep and superficial flexor muscles. Just proximal to the wrist the nerve is divided into a larger palmar cutaneous branch and a smaller median nerve branch that passed under the transverse carpal ligament (Figures 4A–D). On the left, the nerve showed a slight focal hypoechogenic swelling under the carpal ligament (which may have indicated some degree of entrapment), and the nerve then became indistinct in the rudimentary palm area, while on the right (where a hypoplastic thumb was present) two digital branches were visible in the palm area and a recurrent motor nerve for the thumb was seen between the thenar muscles.

The ulnar and superficial radial nerves followed a regular anatomic course bilaterally from the forearm to the hand, with the ulnar nerve having a slightly smaller CSA when compared to normal values for the patients' age and sex. At wrist level, the ulnar nerve on the right ran through an area recognizable as the normal ultrasound configuration of Guyon's channel, to split into two branches that ran from the rudimentary palm of the hand to form the radial and ulnar digital branches of the single ulnar finger. On the left, the ulnar nerve passed a rudimentary entrance to Guyon's channel from which two branches emerged that ran in the rudimentary palm of the hand toward the single digit. The left and right superficial radial nerve branched out over the dorsum of the thumb and ended in different small end branches in the subcutis in this region.

## Discussion

Our high-resolution nerve ultrasound study of these bilateral cleft hands showed nerve sizes and anatomical nerve courses that fit exactly with the presence of the remaining anatomical elements. This suggests that the peripheral nerve innervation in cleft hands can be expected to reflect and follow the presence and course of the remaining target muscles and skin innervation areas. The severe caliber reduction of the median nerve proper in the arm and forearm fits this hypothesis. In addition to the missing intrinsic hand muscles, is it known that the large majority of axons in the upper limb nerves are sensory axons (9), the lack of a part of the palm of the hand and the missing digits will obviate the need for many of these fibers as there is no skin area to innervate.

In the presented patient there is one finger on the left side and one finger with a hypoplastic thumb on the right side. In terms of classification, it needs to be differentiated from ulnar longitudinal deficiency or an oligodactylous type of symbrachydactyly. As the forearm bones, both are equally formed it is more likely not to be an ulnar longitudinal deficiency. Symbrachydactyly can present in the form of an oligodactylous type (10), but it is mostly unilateral and sporadic, without further organ anomalies. The cleft hand syndrome is generally bilateral and of a genetic origin and is often associated with concomitant lower extremity deformities, especially cleft foot malformations. The disorder is bilateral in 56% of cases. Incidence is around 1 in 175,000 births (11). In our patient the third digit on the right foot was hypoplastic. The diagnosis of cleft hand syndrome seems therefore the most logical. Furthermore, malformations in consanguineous offspring are very variable as also is evident in the patient described above.

Our innervation pattern findings are most likely explained by the reciprocal neuro-muscular-bone interaction necessary to correctly guide the embryogenesis of peripheral nerves (12). Embryologically, the appearance of the hand is defined between 48-and 56 days of gestational age. Peripheral nerve formation normally follows targeted muscle growth, with a complex and partially undefined neuromuscular interaction (13). The exclusive innervation of the abnormal ulnar finger by the ulnar nerve may support its embryological origin as a “fifth” finger.

The anatomical description provided here can contribute useful information to correctly guide surgical-reconstructive management, for instance, if a vascular composite transfer is planned like a toe-to-hand transfer. Nerve ultrasound can help in the surgical planning if and where nerve connections can be made. Nerve ultrasound could also help guide other diagnostic or therapeutic techniques such as electrodiagnostic testing or selective peripheral nervous system (PNS) anesthetic blocks in patients with such complex anatomic abnormalities. We hope this report will encourage the use of nerve imaging in the workup of such patients. At the same time, we realize that this high-resolution nerve ultrasound requires training and specific operator expertise to be available. Alternatively, the innervation of these complex anatomic malformations can also be assessed with MR neurography and/or MR tractography when available. Our anatomical findings offer new insights into the anatomy of this rare malformation and may be useful for future pathophysiological studies.

## Data Availability Statement

The raw data supporting the conclusions of this article will be made available by the authors, without undue reservation.

## Ethics Statement

Ethical review and approval was not required for the study on human participants in accordance with the local legislation and institutional requirements. The patients/participants provided their written informed consent to participate in this study. Written informed consent was obtained from the individual(s) for the publication of any potentially identifiable images or data included in this article.

## Author Contributions

SH performed the clinical assessment and oversaw the conventional radiologic studies. PF and NA performed the ultrasound measurements. NA provided the figures. All authors contributed to writing the manuscript draft.

## Conflict of Interest

The authors declare that the research was conducted in the absence of any commercial or financial relationships that could be construed as a potential conflict of interest.

## Publisher's Note

All claims expressed in this article are solely those of the authors and do not necessarily represent those of their affiliated organizations, or those of the publisher, the editors and the reviewers. Any product that may be evaluated in this article, or claim that may be made by its manufacturer, is not guaranteed or endorsed by the publisher.
